# Local delivery of accutox^®^ synergises with immune-checkpoint inhibitors at disrupting tumor growth

**DOI:** 10.1186/s12967-024-05340-2

**Published:** 2024-06-03

**Authors:** Jean Pierre Bikorimana, Nehme El-Hachem, Jamilah Abusarah, Marina Pereira Gonçalves, Roudy Farah, Gabrielle A. Mandl, Sebastien Talbot, Simon Beaudoin, Daniela Stanga, Sebastien Plouffe, Moutih Rafei

**Affiliations:** 1 Department of Microbiology, Infectious Diseases and Immunology, Université de Montréal, 2900 Edouard-Montpetit, Montréal, QC H3T 1J4 Canada; 2 Pediatric Hematology-Oncology Division, Centre Hospitalier Universitaire Sainte-Justine Research Centre, Montreal, QC Canada; 3 AI Branch, Bio2Cure Inc, Montreal, QC Canada; 4 Department of Pharmacology and Physiology, Université de Montréal, Montreal, QC Canada; 5 Department of Molecular Biology, Université de Montréal, Montréal, QC Canada; 6 Department of Biomedical and Molecular Sciences, Queen’s University, Kingston, ON Canada; 7 Research and Development unit, Defence Therapeutics Inc., Montreal, QC Canada

**Keywords:** AccuTOX^®^, Cancer immunotherapy, Immunogenic cell death, Reactive oxygen species, Endosomal escape, Antigen presentation, Immune-Checkpoint inhibitor

## Abstract

**Background:**

The Accum^®^ platform was initially designed to accumulate biomedicines in target cells by inducing endosomal-to-cytosol escape. Interestingly however, the use of unconjugated Accum^®^ was observed to trigger cell death in a variety of cancer cell lines; a property further exploited in the development of Accum^®^-based anti-cancer therapies. Despite the impressive pro-killing abilities of the parent molecule, some cancer cell lines exhibited resistance. This prompted us to test additional Accum^®^ variants, which led to the identification of the AccuTOX^®^ molecule.

**Methods:**

A series of flow-cytometry and cell-based assays were used to assess the pro-killing properties of AccuTOX^®^ along with its ability to trigger the production of reactive oxygen species (ROS), endosomal breaks and antigen presentation. RNA-seq was also conducted to pinpoint the most prominent processes modulated by AccuTOX^®^ treatment in EL4 T-cell lymphoma. Finally, the therapeutic potency of intratumorally-injected AccuTOX^®^ was evaluated in three different murine solid tumor models (EL4, E0771 and B16) both as a monotherapy or in combination with three immune-checkpoint inhibitors (ICI).

**Results:**

In total, 7 Accum^®^ variants were screened for their ability to induce complete cell death in 3 murine (EL4, B16 and E0771) and 3 human (MBA-MD-468, A549, and H460) cancer cell lines of different origins. The selected compound (hereafter refereed to as AccuTOX^®^) displayed an improved killing efficiency (~ 5.5 fold compared to the parental Accum^®^), while retaining its ability to trigger immunogenic cell death, ROS production, and endosomal breaks. Moreover, transcriptomic analysis revealed that low dose AccuTOX^®^ enhances H2-K^b^ cell surface expression as well as antigen presentation in cancer cells. The net outcome culminates in impaired T-cell lymphoma, breast cancer and melanoma growth in vivo especially when combined with anti-CD47, anti-CTLA-4 or anti-PD-1 depending on the animal model.

**Conclusions:**

AccuTOX^®^ exhibits enhanced cancer killing properties, retains all the innate characteristics displayed by the parental Accum^®^ molecule, and synergizes with various ICI in controlling tumor growth. These observations will certainly pave the path to continue the clinical development of this lead compound against multiple solid tumor indications.

**Graphical Abstract:**

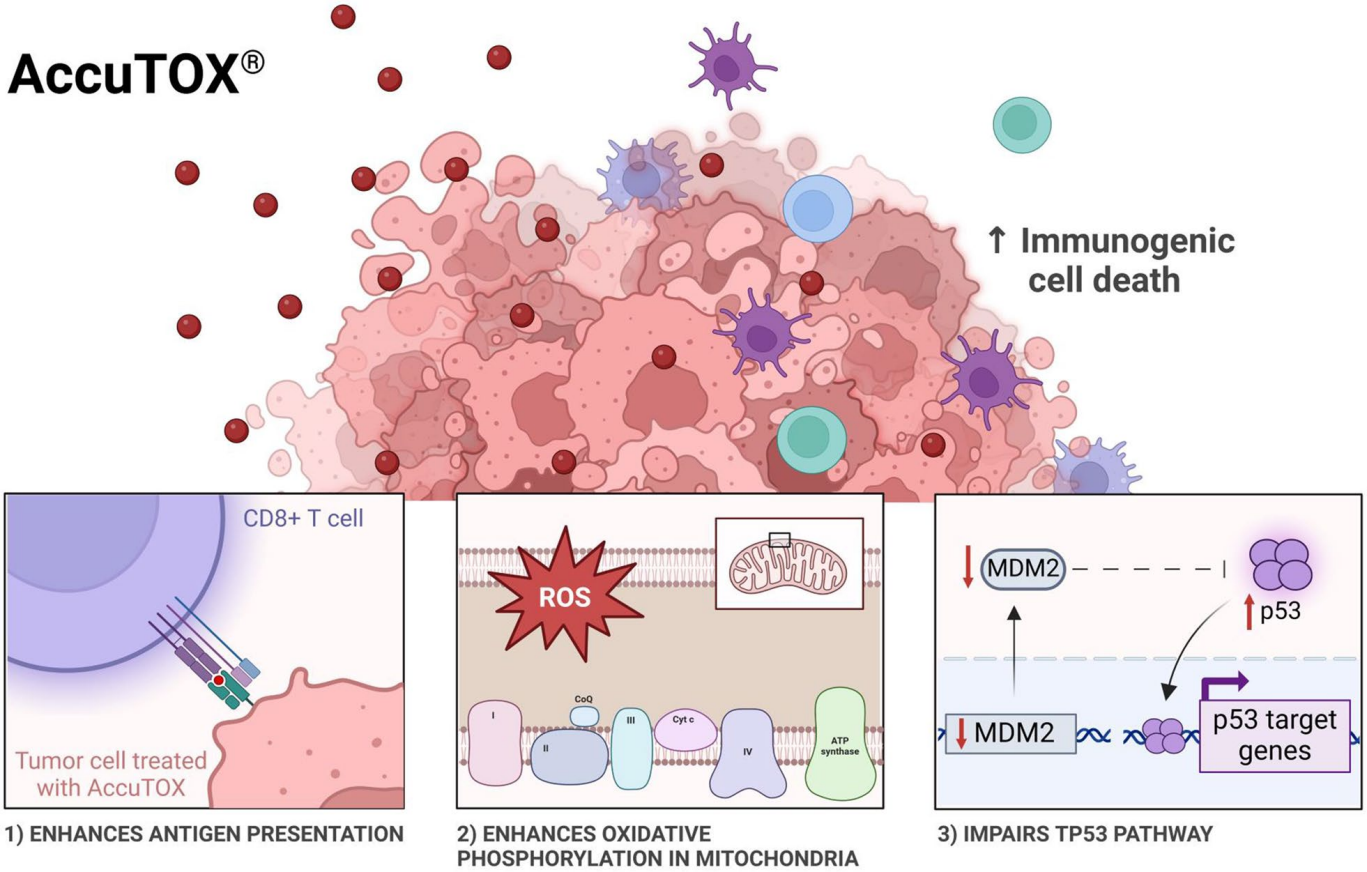

**Supplementary Information:**

The online version contains supplementary material available at 10.1186/s12967-024-05340-2.

## Background

Antibody–drug conjugates (ADCs) are designed to exploit the targeting specificity of monoclonal antibodies (mAb) to deliver cytotoxic chemotherapeutic agents into cancer cells [1, 2]. This approach reduces collateral damage to healthy tissues while improving treatment outcomes [1, 3]. For instance, the mAb Trastuzumab, which was clinically used to target the human epidermal growth factor receptor 2 (HER2) on breast cancer, elicited medium therapeutic effects on patients [4]. This led to further development of the mAb, whereby the anti-microtubule agent DM1 was conjugated as a payload on Trastuzumab (aka T-DM1) [2]. The use of this ADC improved the clinical response of the “naked” mAb as it allowed DM1 delivery in target tumor cells [5]. Despite these encouraging results, several challenges remain, hindering the effectiveness of T-DM1 and other ADCs. One of the most prominent barriers includes the emergence of resistance due to endosomal entrapment and/or recycling of the ADC/payload to plasma membrane [5, 6]. To avoid endosomal entrapment and to improve the bioaccumulation of these payloads in target cells, Lacasse et al*.* engineered a molecule named Accum^®^ [7]. This lipopeptide, which is composed of a cholic acid fused to a nuclear localization signal (NLS), hijacks specific cellular transport pathways by facilitating endosomal escape [7]. More specifically, Accum^®^ selectively disrupts endosomal membranes via ceramide formation, resulting in membrane destabilisation/disruption allowing molecules to leak into the cytosol. The NLS then targets the delivered payload to the nucleus causing genotoxic effects [7]. This is further exemplified with the use of 7G3-Accum^®^ and A14-Accum^®^, two antibodies targeting IL-3Ra in TF-1a leukemic cells and IL-5Ra in muscle invasive bladder cancer, respectively [8, 9]. In both cases, Accum^®^ bio-conjugation increased mAb accumulation compared to the “naked” antibody [8, 9]. Similarly, Accum^®^ bio-conjugation to T-DM1 enhanced its cytotoxic effect by 18-fold as previously shown using the HER2-positive SKBR3 breast cancer cell line [7–10].

The improved bioaccumulation of mAbs in target cells instilled the idea of evaluating the possible use of the Accum^®^ technology in the context of protein-based vaccination. [10–12] Accum^®^ bio-conjugation onto antigens provided double benefit as it: (i) protected the antigen from excessive degradation within the endosome, and (ii) enabled antigen leakage into the cytoplasm where it was effectively processed (as an almost intact protein) by the proteasomal complex [11, 12]. Antigen-Accum^®^ bio-conjugation therefore allowed for improved preservation and presentation of immunogenic peptides on the surface of antigen presenting cells, which is essential for priming CD8 T-cell to trigger an effective anti-tumoral response [11–13]. In support of this notion, tumor growth was impaired when animals underwent therapeutic vaccination using dendritic cells (DCs) pulsed with Accum^®^-linked antigens. [11] Another example is the use of Accum^®^ conjugated onto the human papilloma virus E7 oncoprotein as a protein-based therapeutic vaccine, which not only protected the host from tumor establishment (prophylactic vaccination) but was also capable of impairing cervical cancer growth in mice when used as a therapeutic vaccine [12].

While investigating the use of Accum^®^ in vaccine engineering, a novel function was uncovered for the unconjugated molecule. More specifically, Accum^®^ triggered immunogenic cell death in various murine tumor cell lines with marked endosomal damage and increased production of reactive oxygen species (ROS) [14]. When tested in vivo, intra-tumoral (IT) injection of unconjugated Accum^®^ successfully inhibited tumor growth in mice with pre-established EL4 T cell lymphoma, an effect that was dose dependent and reliant on T-cell activity as well as immune-check point inhibitors (ICI) [14]. Along this line of thought, the original Accum^®^ molecule was further modified to create a second-generation entity, named AccuTOX^®^. Studies aimed at characterizing its therapeutic potential revealed that AccuTOX^®^ exhibits powerful pro-killing properties and synergizes with different ICI at controlling cancer growth.

## Methods

### Mouse strains

All female and male C57BL/6 mice were aged 6–10 weeks and were purchased from Charles River (Montreal, QC, Canada). Animals were housed in a pathogen-free environment at the Institute for Research in Immunology and Cancer animal facility (Université de Montréal). The animal protocol (#22–065) used in this study was approved by the Animal Care Committee (CDEA) of Université de Montréal. All in vivo studies were conducted on animals of similar age (⁓8–9 weeks old).

### Cell lines

The EL4, EG.7, and B16 tumor cells were kindly provided by Dr. Jacques Galipeau (University of Wisconsin-Madison, WI, USA). The E0771 breast cancer cell line was a kind gift from Dr. John Stagg (Université de Montréal, QC, Canada). The MBA-MD-468, A549 and H460 were a kind gift from Dr. Audrey Claing (Université de Montréal, QC, Canada). The B3Z cell line was a kind gift from Dr. Etienne Gagnon (Université de Montréal, QC, Canada). EL4 and H460 tumor cells were cultured in RPMI 1640 supplemented with 2 g/L Glucose, 10% FBS, and 50 U/mL Penicillin–Streptomycin. The B3Z and EG.7 cells were cultured in RPMI 1460 supplemented with 10% FBS, 50 U/mL Penicillin–Streptomycin, 2 mM L-glutamine, 10 mM HEPES, 1 mM Sodium Pyruvate, and 0.5 mM β-Mercaptoethanol. E.G7 were kept under selection using 0.4 mg/mL of G418. B16, MBA-MD-468, E0771 and A549 tumor cells in addition to primary mesenchymal stromal cells (MSCs) were cultured in DMEM supplemented with 10% FBS and 50 U/ml Penicillin–Streptomycin. All cells were maintained at 37 °C in a 5% CO_2 _incubator. All cell culture media and reagents were purchased from Wisent Bioproducts (St-Bruno, QC, Canada).

### Antibodies and reagents

The H2-K^b^ antibody was purchased from BD Biosciences (San Jose, CA, USA). The anti-PD-1 antibody (clone RMP1-14) for in vivo studies was purchased from Assay Genie (Dublin, Ireland). The anti-CTLA4 (clone 9D9), and anti-CD47 (clone MIAP301) used for in vivo studies were purchased from BioXCell (Lebanon, NH, USA). The calreticulin primary antibody (ab2907) was purchased from Abcam (Toronto, ON, Canada). The ENLITEN-ATP kit was purchased from Promega (Madison, WI, USA). The MitoSOX^™^, DHE and MitoTEMPO reagents were purchased from Thermofisher Scientific (Markham, ON, Canada) and used according to manufacturer’s instructions. Cytochrome C (Cyt-C), N-acetylcysteine (NAC), and α-tocopherol were purchased from Millipore-Sigma (Burlington, MA, USA). The Annexin-V^+^ staining kit was purchased from Cedarlane laboratories (Burlington, ON, Canada). The RNeasy^®^ mini kit was purchased from QIAGEN (Toronto, ON, Canada). The AccuTOX^®^ and its derivatives were synthesized as previously described [9].

### Determining the AccuTOX^®^ IC_50_

To determine the AccuTOX^®^ IC_50_, EL4 cells were seeded at a density of 5 × 10^4^ cells/well in a round bottom 96-well plate (final volume of 250 µl). Cells were treated overnight with different concentrations of AccuTOX^®^ (0, 0.7, 1.5, 2.8, 5.6, 11.3, 22, 45. 91, 181, 262 and 500 µM). The following day, cells were washed with PBS and stained with Annexin-V^+^ according to manufacturer’s instructions. The signal was detected using BD FACS Diva on CANTOII. Signal analysis was done using FlowJo and IC_50_ calculated using the GraphPad Prism 10 software.

### Apoptosis analysis

Apoptosis analysis was conducted by flow-cytometry as previously reported [14]. Briefly, target cells (EL4, B16, E0771, MBA-MD-468, A549, and H460) were first treated with 33 µM of AccuTOX^®^ (unless otherwise stated) overnight then washed twice with PBS containing 2% FBS. Treated cells were then re-suspended in Annexin-V^+^ staining buffer before reagent staining according to manufacturer’s instructions. Fifteen minutes later, stained cells were washed with PBS containing 2% FBS prior to signal detection using BD FACS Diva on CANTOII, followed by analysis using FlowJo.

### Assessment of in vitro immunogenic cell death (ICD)

To obtain conditioned media (CM), 5 × 10^5^ EL4 cells were seeded in 24-well plates in culture media for 24 h followed by treatment with 16.58 µM of AccuTOX^®^ overnight. The IC_50_ dose was used to detect ICD changes without inducing complete cell death. The ATP concentration in the CM was quantified using the ENLITEN-ATP kit. Briefly, 100 μL of CM was transferred to 96-well opaque plates. Then 100 μL of reconstituted luciferase/luciferin reagent was added to each well followed by measurement of luciferase using a luminescence microplate reader (Fusion V.3.0). As for calreticulin exposure, treated cells were harvested and cell surface calreticulin exposure was measured by flow-cytometry (n = 6/condition). The calreticulin primary antibody was added to cells for 20 min at 4 °C, followed by washing with flow cytometry buffer (PBS + 2% FBS), then stained with goat anti-rabbit Alexa647 secondary antibody (Life Technologies) for an additional 20 min at 4 °C. The samples were washed twice and resuspend in flow cytometry buffer. The signal was captured then data analyzed using BD FACS Diva on CANTOII, and FlowJo, respectively.

### RNA extraction and sequencing

Briefly, total RNA was isolated from 10^6^ AccuTOX^®^-treated EL4 cells (30 min treatment) using the RNeasy^®^ mini kit (QIAGEN, Toronto, ON, Canada) according to manufacturer’s instructions. Library preparation and sequencing were performed at the Institute for Research in Immunology and Cancer’s Genomics Platform as previously described [15].

### Bioinformatics analysis

All Fastq files were aligned to GRCm38 (mouse genome Ensemble release 102) with STAR (v2.7). Raw reads mapping to genomic features (summarized per gene) were extracted with featureCounts (strand specific option). Mouse genes were mapped to corresponding Human orthologs. Expression matrices were filtered, genes with very low counts were removed and protein-coding genes were kept for further analyses. Accum^®^-treated cells were contrasted to the control group with DESeq2 to generate a ranked list of differentially expressed genes based on the log2 fold change with a significance threshold is set to 5% after p-value adjustment with the Benjamini–Hochberg method to control for false positives among differentially expressed genes. All custom scripts were written in R programming and statistical language. Plots and heatmaps were made with ggplot2 and pheatmap R packages.

### Evaluating the role of ROS in AccuTOX^®^-induced cell death

To assess the possible role played by ROS in AccuTOX^®^-induced cell death, EL4 cells were first treated with 10 mM of NAC, 800 μM of α-tocopherol, or 10 μM of MitoTEMPO for 1 h [16, 17]. Following the incubation period, 33 µM of AccuTOX^®^ was added and cell death was assessed the following day by Annexin-V^+^ staining as detailed above, followed by signal detection using BD FACS Diva on CANTOII and then analyzed using FlowJo.

### Assessment of endosomal escape

To evaluate endosomal escape, 10^5^ EL4 or primary MSCs were first supplemented with 10 mg/mL exogenous Cyt-C for 6 h at 37 °C in the presence or absence of AccuTOX^®^ (using the IC_50_ dose) [18]. Following the incubation period, treated cells were washed with ice-cold PBS, then stained for Annexin-V^+^ according to manufacturer’s instructions prior to analysis using BD FACS Diva on CANTOII as detailed above.

### Antigen presentation assay

The antigen presentation assay was conducted in a 24-well plate. Briefly, 5 × 10^5^ EG.7 cells were treated with ascending doses of AccuTOX^®^ (1, 4, 8, 16, and 32 μM) overnight. On the following day, treated cells were washed with PBS, then 5 × 10^5^ B3Z cells were added per well. The co-culture was incubated for 17–19 h. The media was then removed, and the cells washed once with PBS. Cells were then lysed using lysis buffer (tris base, CDTA, glycerol and triton X-100) and shaken for 20 min at room temperature. Cell lysate was then incubated with a CPRG solution (containing CPRG, disodium phosphate, monosodium phosphate, potassium chloride, magnesium sulfate) and protected from light for 24 h at 37 °C [19]. The optical density signal was detected at a wavelength of 570 nm using a SynergyH1 microplate reader (Biotek, Winooski, VT, USA).

### In vivo treatment studies

For in vivo studies, mice (n = 5–10/group depending on the experiment) were subcutaneously (SC) implanted with EL4, E0771 or B16 cells (0.5 × 10^6^ cells /injection). Three to 4 days later, palpable tumors were injected with 8 mg/kg of AccuTOX^®^ for a total of 6 IT injections (once every 48 h). Control animals (n = 5/group) were injected with equivalent volumes of PBS. For Accum^®^-related studies, animals received the Accum^®^ compound (16 mg/kg—to maintain equimolar concentrations with AccuTOX^®^) following the same dosing schedule used for AccuTOX^®^. All used ICIs were administered at 200 μg per injection and delivered via the intraperitoneal (IP) route 3 times per week for two consecutive weeks (total of 6 injections) [14]. All vaccinated animals were monitored for up to 6 weeks. Tumor size and animal survival for the above listed in vivo studies were followed thereafter until reaching endpoints (ulceration or a tumor volume ≥ 1000 mm^3^). Male and female mice (n = 10/group) used for toxicology studies were assessed for: (i) inflammation at site of SC injection, (ii) overall activity, (iii) condition of their fur, (iv) body posture, and (v) weigh loss over time. Mice were giving an arbitrary score of 0–4, with 0 reflecting no noticeable sign versus 4, a score representing moribund animals. For studies investigating the AccuTOX^®^-enhanced immunogenicity of EG.7 tumors, 0.5 × 10^6^ EG.7 cells were treated in vitro with 1 μM of AccuTOX^®^ overnight before SC transplantation in both male and female mice (n = 10/group). Vehicle/media-treated EG.7 cells were used as comparative controls. Animals were then monitored for tumor growth overtime.

### Statistical analysis

Depending on the study, *p-values* were calculated using the student’s t-test, one-way analysis of variance (ANOVA) or the log-rank test using GraphPad Prism 10. Results are represented as means with standard deviation (S.D.) error bars, and statistical significance is represented with asterisks: *P < 0.05, **P < 0.01, ***P < 0.001.

## Results

### AccuTOX^®^: an Accum^®^ variant with enhanced killing properties

**Fig. 1:**
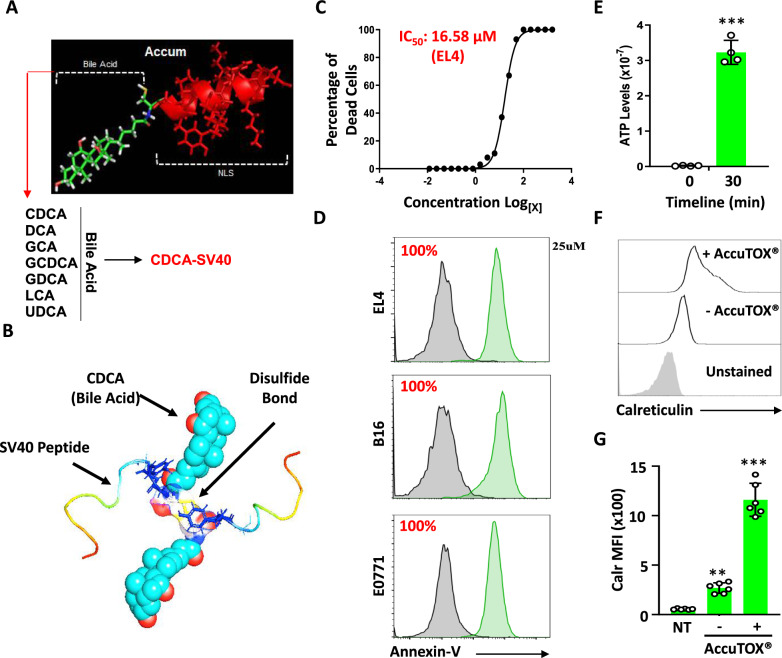
AccuTOX^®^ triggers immunogenic cell death. **A** Schematic diagram outlining the strategy used for the generation of the Accum^®^ variants. **B** A predicted 3D model depicting the structure of the AccuTOX^®^. **C** A killing dose–response curve conducted on the EL4 lymphoma cell line to identify the IC_50_ dose. **D** Representative flow-cytometry analysis assessing Annexin-V^+^ using three different cancer cell lines (EL4, B16 and E0771) following an overnight in vitro treatment with AccuTOX^®^ (33 μM). Control cells are shown by gray histograms. **E** Assessment of ATP secretion levels overtime in response to AccuTOX^®^ treatment (33 μM). **F** Flow-cytometry analysis of Calreticulin^+^ cells in response to AccuTOX^®^ treatment (33 μM). **G** The mean fluorescent intensity (MFI) of the Calreticulin signal shown in panel F. For panels G, n = 6/group with ***P < 0.001. All experiments shown in this panel were repeated 3 times

We have recently reported that unconjugated Accum^®^ exerts anti-tumoral activities both in vitro and in vivo [14]. However, the pro-apoptotic ability of the parental Accum^®^ molecule was inconsistent as it triggered cell death with different efficiencies on cancer cells, with B16 melanoma being the most resistant [14]. We thus engineered a family of 7 Accum^®^ variants by interchanging the original cholic acid with 7 other bile acids (CDCA, DCA, GCA, GCDCA, GDCA, LCA or UDCA) while keeping the SV40 nuclear localization signal (NLS) intact (Fig. 1A). Compared to the original Accum^®^ molecule, treatment of various murine and human cancer cell lines revealed consistent and complete killing using the CDCA-SV40 variant (Suppl. Figure 1A). To further optimize the killing potency of this variant, we next asked whether additional modifications could affect the activity of the CDCA-SV40 variant. We thus: (i) mutated the cysteine residue between the bile acid and the SV40 peptide into an alanine (C → A), (ii) tested a dimer of the CDCA-SV40 molecule (CDCA-C-SV40 NLS)_2_, or (iii) added a cleavable (GSH) or uncleavable (MPA) moiety on the cysteine residue of CDCA-SV40. Using the EL4 cell line as a working model, we found that the CDCA-SV40 dimer (aka AccuTOX^®^—Fig. 1B) triggers potent killing whereas mutating or tagging the cysteine residue impairs the molecule’s ability to promote cell death (Suppl. Figure 1B). Based on these observations, we next conducted an AccuTOX^®^ killing curve using the EL4 cell line and identified the IC_50_ dose to be 16.58 μM (Fig. 1C). When tested on 3 different tumor cell lines using the IC_100_ dose, AccuTOX^®^ triggered complete cell death (Fig. 1D). Akin to the original Accum^®^ molecule, AccuTOX^®^ was also capable of eliciting ICD as shown by the secretion of ATP in the supernatant of AccuTOX^®^-treated EL4 cells (Fig. 1E) as well as the cell surface increase in calreticulin intensity (Fig. 1F, G). In sum, we engineered an Accum^®^ variant endowed with enhanced cancer killing properties while retaining its original ability at inducing ICD.

### Administration of unconjugated AccuTOX® as a combination therapy with ICI delays the growth of pre-established solid tumors

**Fig. 2:**
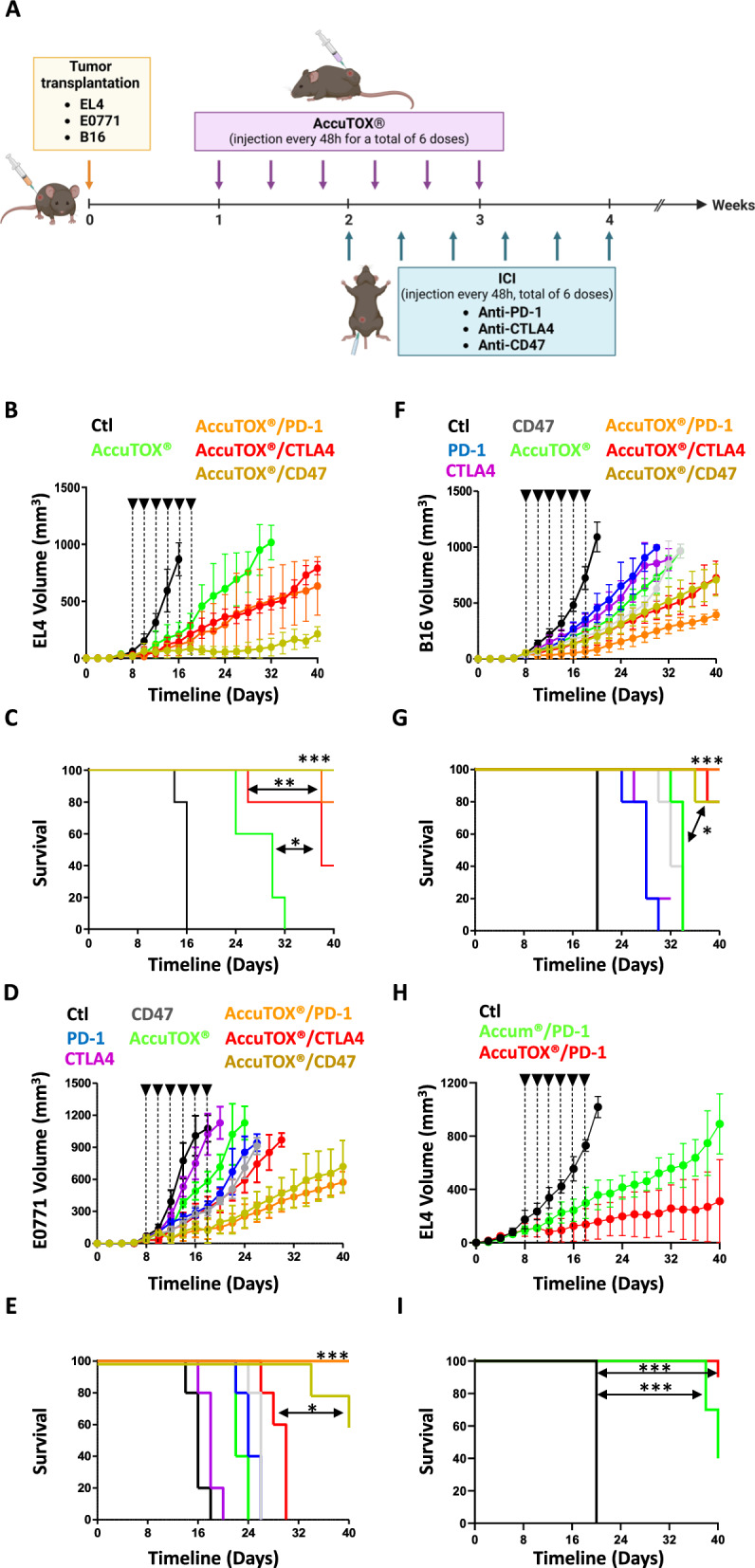
AccuTOX^®^ synergises with ICI at impairing tumor growth. **A** Schematic diagram showing the approach used for the in vivo studies using three cancer models. **B** Assessment of EL4 tumor volume overtime when combined with anti-PD-1, anti-CTLA4 or anti-CD47. The color code is as follows: control (black), AccuTOX^®^ (green), AccuTOX^®^ + anti-PD-1 (orange), AccuTOX^®^ + anti-CTLA-4 (red), AccuTOX^®^ + anti-CD47 (yellow). **C** Kaplan–Meier survival curve for the experiment in panel B. **D** Assessment of E0771 tumor volume overtime when combined with anti-PD-1, anti-CTLA4 or anti-CD47. The color code is as follows: control (black), anti-PD-1 (green), anti-CLTA-4 (purple), anti-CD47 (gray), AccuTOX^®^ (blue), AccuTOX^®^ + anti-PD-1 (orange), AccuTOX^®^ + anti-CTLA-4 (red) and AccuTOX^®^ + anti-CD47 (yellow). **E** Kaplan–Meier survival curve for the experiment in panel D. **F** Assessment of B16 tumor volume overtime when combined with anti-PD-1, anti-CTLA4 or anti-CD47. The color code is as follows: control (black), anti-PD-1 (green), anti-CLTA-4 (purple), anti-CD47 (blue), AccuTOX^®^ (gray), AccuTOX^®^ + anti-PD-1 (orange), AccuTOX^®^ + anti-CTLA-4 (red) and AccuTOX^®^ + anti-CD47 (yellow). **G** Kaplan–Meier survival curve for the experiment in panel F. **H)** Assessment of B16 tumor volume overtime in response to Accum^®^ (green) or AccuTOX^®^ (red) in combination with anti-PD-1. **I** Kaplan–Meier survival curve for the experiment in panel H. For all in vivo panels shown in panels B-G, n = 5/group with *P < 0.05, **P < 0.01, and ***P < 0.001. For the in vivo experiment shown in panels H-I, n = 10/group. The in vivo experiments were repeated twice

Prior to assessing the in vivo tumor-killing property of unconjugated AccuTOX^®^, we first analyzed the expression profile of the most studied immune-checkpoints on the surface of EL4 T-cell lymphoma, E0771 breast cancer and B16 melanoma. As shown in Suppl. Figure 2, all three cell lines were negative for CTLA-4 expression but displayed substantial levels of the CD47 “don’t eat me” signal. PD-L1 expression, on the other hand, was only detected on the surface of E0771 and B16 with a minor (insignificant) signal on the surface of EL4 cells. With these data in hand, we next tested the therapeutic potency of IT-injected AccuTOX^®^ (8 mg/kg) as a monotherapy or in combination with these ICIs in immunocompetent C57BL/6 mice transplanted with one of the three syngeneic cancer models (Fig. 2A). For this study, animals with pre-established tumors received an AccuTOX^®^ injection every 48 h (3 per week) for a total of 6 injections, while ICI were administered starting at week 2, with the second AccuTOX^®^ dosing, (ICI controls were not used in the EL4 model as they were previously shown to be inert) [14]. Consistent with the ICI analysis on the surface of cancer cells, AccuTOX^®^ combined with anti-CD47 (yellow line) was substantially superior than AccuTOX^®^ alone (green line) at inhibiting EL4 T-cell lymphoma growth (Fig. 2B). In fact, 100% of animals undergoing this combination therapy survived by day 40 post-tumor transplantation (Fig. 2C). Similar tumor growth patterns were observed in the E0771 breast cancer model when AccuTOX^®^ was combined with anti-PD-1 (orange line) or anti-CD47 (yellow line—Fig. 2D) with a final survival rate of 60 and 100% respectively (Fig. 2E). As for the B16 melanoma model, AccuTOX^®^ was mostly efficient when combined with anti-PD-1 (orange line) followed by both anti-CTLA-4 (red line) and anti-CD47 (yellow line—Fig. 2F) with a survival rate of 100% for the anti-PD-1 versus 80% for anti-CTLA-4 or anti-CD47 (Fig. 2G). Since the main objective of the study herein is to engineer a second-generation Accum^®^ molecule with enhanced anti-tumoral activity, we next conducted a head-to-head comparison of Accum^®^ versus AccuTOX^®^ (injected at equimolar concentrations) using the B16 melanoma model. As shown in Fig. 2H, AccuTOX^®^ combined to anti-PD-1 was superior at impairing tumor growth with a survival rate of 90% compared to 40% with the use of the Accum^®^ anti-PD-1 combination (Fig. 2I). Altogether, our results could be summarized in two ways. First, AccuTOX^®^ can be used against different solid tumors. Second, the compound synergizes with different ICI (depending on the tumor model) at impairing tumor growth.

### AccuTOX^®^ is effective in both sexes and exhibits a relatively low toxicity profile

**Fig. 3:**
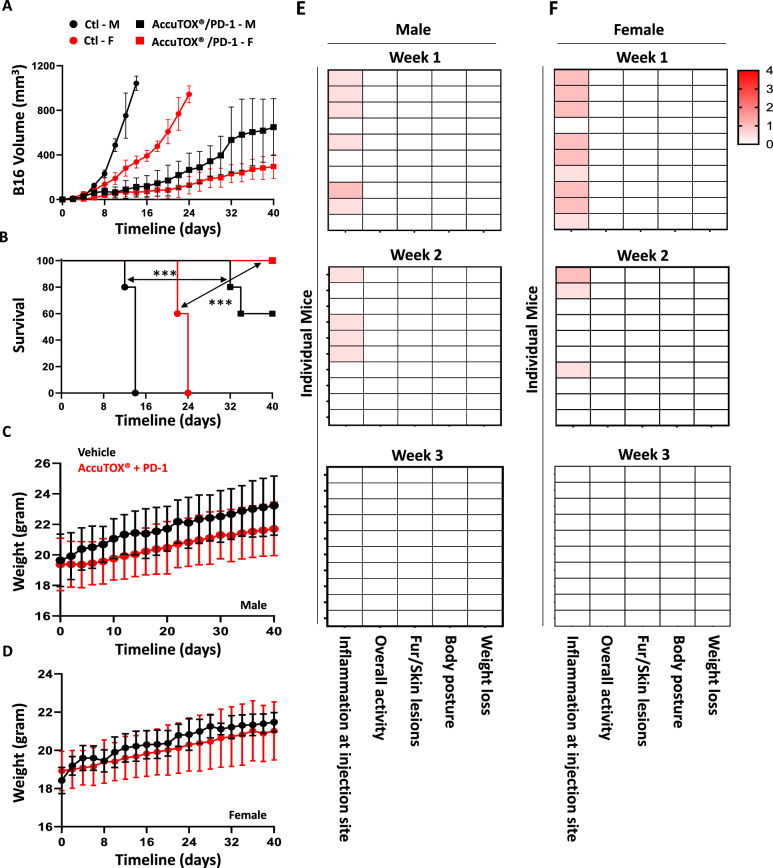
Sex-based therapeutic potency and toxicity profiling. **A** Comparing the potency of the AccuTOX^®^/PD-1 combination in male and female mice transplanted with B16 tumors. The color code is as follows: male control (black circles), AccuTOX^®^/PD-1 in male mice (black squares), female control (red circles), AccuTOX^®^/PD-1 in female mice (red squares). **B** Kaplan–Meier survival curve for the experiment in panel A. **C** Weight loss assessment in male mice undergoing AccuTOX^®^/PD-1 treatment following B16 transplantation. **D** Same as panel C but on female mice. **E** Analysis of clinical signs in male mice for the experiments shown in panels A and C. **F** Analysis of clinical signs in female mice for the experiments shown in panels A and D. For the in vivo experiment shown in panels A-B, n = 10/group. The in vivo experiments were repeated twice

Since sex is considered a biological variable to account for in pre-clinical research, we next conducted an in vivo study comparing the potency and toxicity profiles of the AccuTOX^®^/PD-1 combination in both male and female mice transplanted with the B16 melanoma. Interestingly, the B16 melanoma grew with a more aggressive pattern in male mice, which may explain the reduced potency of the treatment compared to female counterparts (Fig. 3A). Nevertheless, the AccuTOX^®^/PD-1 combination substantially delayed tumor growth in both animal groups with female mice resulting in a survival rate of 100% versus 60% in male mice respectively (Fig. 3B).

To assess the toxicity profile of the treatment, male and female mice with B16 tumors undergoing AccuTOX^®^/PD-1 treatments were assessed for various toxicological parameters. Besides following animal weights, which did not reflect any losses over the lifetime of the experiment (Fig. 3E, F), we analyzed animals for any signs of discomfort. Although both male and female mice exhibited no signs of skin lesions or ruffled fur, nor unusual body poster, a decrease in overall activity (observed over a period of 5–10 min) was observed, and both sex groups had some signs of local inflammation at the site of injections (scores ranging from 0.5 to 1 on a scale of 4). In fact, most of these inflammatory signs were noticed during the first and second weeks of AccuTOX^®^ administration (Fig. 3E, F). In sum, AccuTOX^®^/PD-1 co-administration induces potent anti-tumoral effects in both male and female mice and does not seem to trigger alarming side effects.

### Molecular profiling of AccuTOX^®^-treated EL4 cells reveals the modulation of pathways relevant to cancer cell death and immune activation

**Fig. 4:**
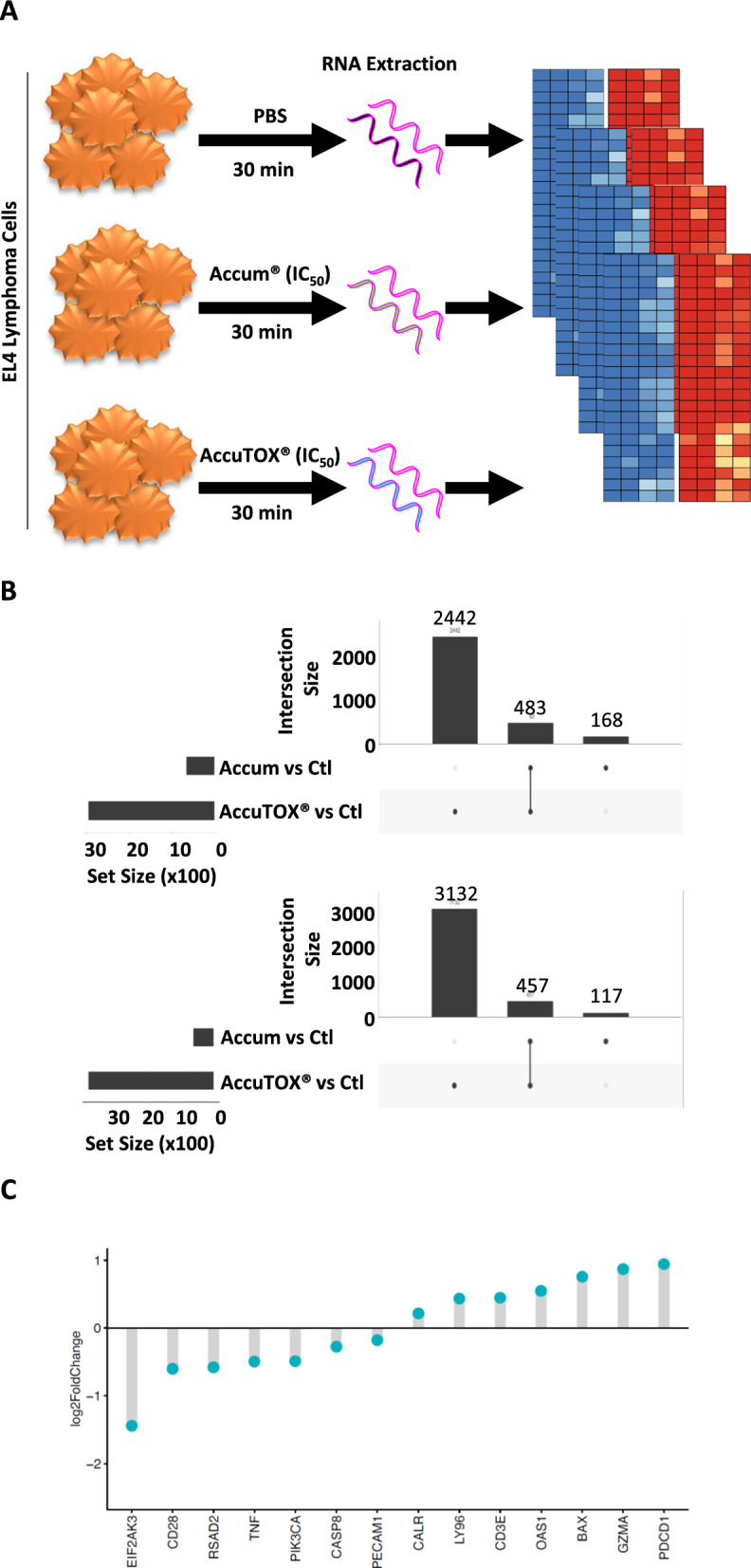
Transcriptomic analysis of AccuTOX®-treated cancer cells. **A** Schematic diagram depicting the experimental design used for the RNA-seq experiment. **B** The upset plots illustrate the count of genes that are either commonly or uniquely regulated, either upregulated (upper panel) or downregulated (lower panel), by Accum^®^ or AccuTOX^®^ in EL4 cells when compared to untreated controls. A 5% adjusted p-value was used to determine the differentially expressed genes. **C** AccuTOX^®^ treatment either induces or downregulates genes associated with ICD. A significance level of 5% was considered based on adjusted p-values

**Fig. 5:**
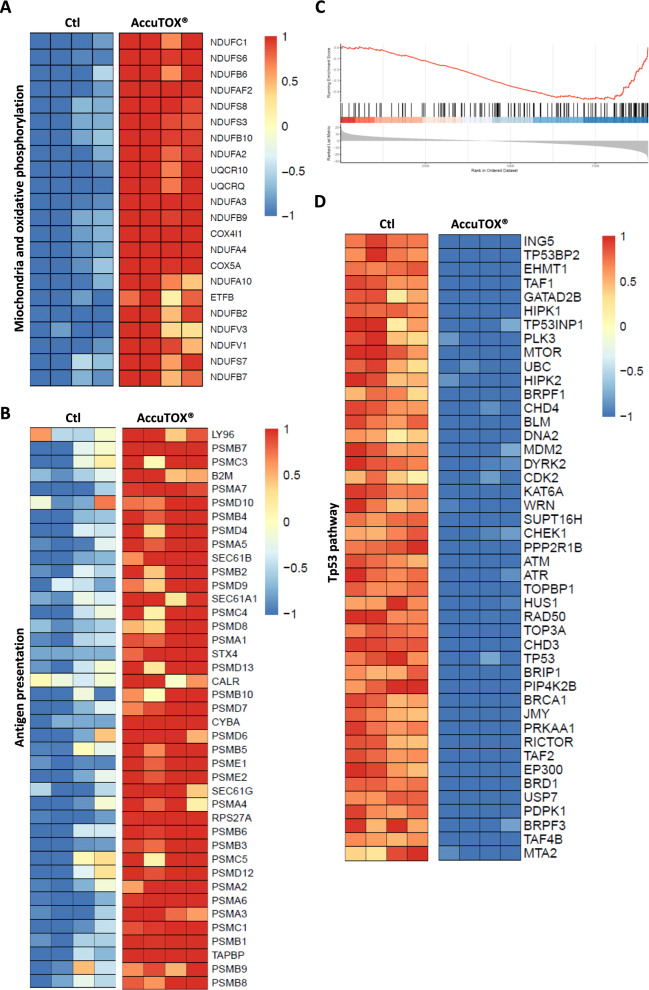
AccuTOX^®^ treatment modulates various molecular processes in cancer cells. **A** The heatmap depicts the upregulated genes associated with mitochondrial oxidative phosphorylation—complex I (Reactome pathways), significantly contributing to the enrichment score in gene set analysis. **B** Similar to A, this heatmap represents the Reactome pathway “antigen presentation”, highlighting upregulated genes that significantly contribute to the enrichment score from gene set analysis. **C** The enrichment plot displays the ranking of genes involved in the regulation of TP53 activity against the ranked list of differentially expressed genes in the AccuTOX^®^ group. The genes are ordered from left to right based on decreasing fold change. **D** The heatmap illustrates the robust repression by AccutOX^®^ of genes associated with the regulation of TP53 activity (Reactome pathways), significantly impacting the expression levels

The fact that AccuTOX^®^ readily affects tumor growth prompted us to investigate changes induced at the molecular level using transcriptomic analyses. We thus compared gene expression of EL4 cells following a 30 min treatment with Accum^®^ or AccuTOX^®^ using the IC_50_ dose to avoid triggering complete cell death (Fig. 4A). As shown in the upper panel of Fig. 4B, AccuTOX^®^ exhibits a broader impact on gene expression as 2442 genes were upregulated in contrast to 168 genes with Accum^®^. Similarly, 3132 genes were downregulated in response to AccuTOX^®^ treatment versus 117 genes with Accum^®^ (lower panel). Besides, AccuTOX^®^ induces the activation of a cluster of genes associated with ICD, including CALR, BAX and GZMA along with a significant overexpression of PD-1 (PDCD1 gene—Fig. 4C). Additional investigations on gene set enrichment revealed common upregulation of genes involved in oxidative phosphorylation and antigen presentation in response to AccuTOX^®^ (Fig. 5A, B). Moreover, AccuTOX^®^ treatment results in the suppression of genes regulating TP53 activity (Fig. 5C, D; Normalized enrichment score = − 1.76, q-value = 0.001). Many of the affected genes in this pathway are crucial for repairing double-strand breaks (ATM, ATR, CHEK1, BRCA1), suggesting that AccuTOX^®^ may induce irreversible damages to target cells. In other words, by shutting down the cellular repair machinery, AccuTOX^®^ is most likely promoting rapid cancer cell death.

### AccuTOX^®^ disrupts cancer cell growth by targeting multiple intracellular facets

**Fig. 6:**
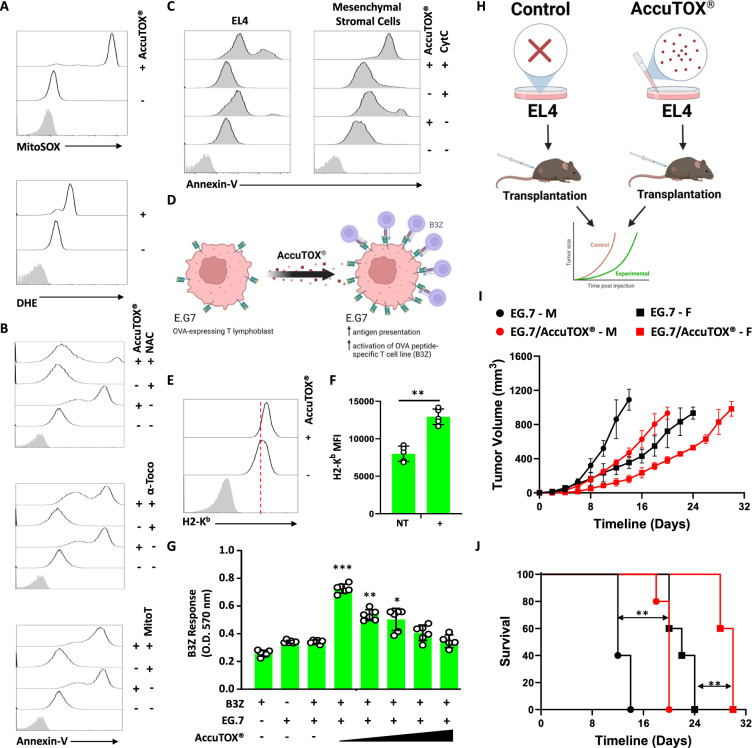
AccuTOX^®^ triggers ROS, disrupts endosomal membranes and enhances antigen presentation in cancer cells. **A** Analysis of ROS production using flow-cytometry-based MitoSOX™/DHE staining of cells treated with 33 μM of AccuTOX^®^. **B** Annexin-V^+^ staining of EL4 cells pre-treated with 5 mM NAC, 800 μM of α-tocopherol or 10 μM MitoTEMPO 1 h prior AccuTOX^®^ treatment using the IC_50_ dose. **C** A representative flow-cytometry analysis assessing Annexin-V in response to AccuTOX^®^ and Cyt-C co-treatment in EL4 cancer cells (left panel) and primary MSCs (right panel). **D** A representative cartoon depicting the antigen presentation assay using the EG.7 system. **E** A representative flow-cytometry analysis of H2-K^b^ on the surface of EG.7 treated with AccuTOX^®^. **F** Quantification of the means fluorescence intensity of the experiment shown in panel E. **G** Quantification of B3Z activation in response to EG.7 pre-treated with ascending doses of AccuTOX^®^ (1, 4, 8, 16, and 32 μM). **H** Schematic diagram depicting the transplantation study using 1 μM AccuTOX^®^-treated EG.7 cells. **I** Volume assessment of control versus in vitro AccuTOX^®^-pretreated EG.7 tumors in male (circles) versus female (square) mice. **J** Kaplan–Meier survival curve for the experiment in panel I. For panels F and G, n = 5/group with *P < 0.05, **P < 0.01, and ***P < 0.001. For panels I and J, n = 10/group with **P < 0.01

The parental Accum^®^ molecule triggers cell death by inducing both ROS production as well as endosomal membrane disruption [14]. Following confirmation that AccuTOX^®^ elicits ROS in EL4 using both MitoSOX™ and DHE staining (Fig. 6A), we next evaluated whether different antioxidants could reverse the killing ability of the compound. Interestingly, only NAC pre-treatment blocked AccuTOX^®^-induced cell death whereas α-tocopherol (to inhibit lipid peroxidation) or mitoTEMPO (to block mitochondrial ROS production) had no noticeable effect (Fig. 6B). Since the Accum^®^ platform is based on endosomal damages, we next evaluated whether AccuTOX^®^ retains this ability by pulsing EL4 cells with Cyt-C admixed with AccuTOX^®^ prior to assessing Annexin-V^+^ as previously shown [14]. If AccuTOX^®^ breaks down endosomal membranes, then Cyt-C diffuses to the cytosol where it can activate caspases, consequently resulting in cell death [14]. Indeed, EL4 treatment with Cyt-C triggered no cell death, as opposed to 100% Annexin-V^+^ positive events obtained in the AccuTOX^®^/Cyt-C group (Fig. 6C—left panel). This effect was not specific to cancer cell lines, as a similar outcome was observed using wild-type primary MSCs as non-cancerous cells (Fig. 6C—right panel).

Besides changes related to ROS production and endosomal breaks, our transcriptomic analysis revealed yet another salient observation highly relevant to ICD and/or stimulation of anti-tumoral immunity. More specifically, several genes related to antigen presentation were upregulated in response to AccuTOX^®^ treatment (Fig. 5B). This implies that AccuTOX^®^ could render cancer cells immunogenic by enhancing the presentation of endogenous intracellular antigens. To test this hypothesis, we treated EG.7 cells (an EL4 cell line modified to express the ovalbumin protein) with the IC_50_ dose of AccuTOX^®^ and assessed antigen presentation of the ovalbumin-derived SIINFEKL peptide (Fig. 6D). Besides detecting a significant increase in H2-K^b^ expression on the cell surface of EG.7 cells (Fig. 6E, F), the antigen presentation assay revealed a substantial enhancement in the activation of the B3Z T-cell line (specific to the SIINFEKL peptide in the context of H2-K^b^) with the highest signal obtained using 1 μM of AccuTOX^®^ (Fig. 6G). To further validate the enhanced immunogenicity of EG.7 triggered by AccuTOX^®^, we conducted an in vivo study where immunocompetent male and female mice were SC transplanted with non-treated versus AccuTOX^®^-treated EG.7 cells (Fig. 6H). Although a delay in the growth of AccuTOX^®^-treated EG.7 was observed in both sexes (Fig. 6I), female recipients exhibited strong tumor delay responses compared to their male counterparts, most likely due to potent female-driven immunity (Fig. 6I, J). These results imply that in addition to enhanced killing, AccuTOX^®^ can stimulate antigen presentation, which could result in the initiation of anti-tumoral immunity.

## Discussion

In response to Accum^®^, cancer cells undergo a variety of intracellular changes characterized by the elevated production of ROS, disruption of endosomal integrity and production of several factors related to ICD [14]. Despite the observed resistance of certain cancer cell types in vitro (e.g. melanoma), IT injection of unconjugated Accum^®^ could delay pre-established EL4 T-cell lymphoma growth when combined with ICIs; a therapeutic effect that seems to depend on dendritic cells as well as CD4 and CD8 lymphocytes [14]. This suggests that Accum^®^ has dual roles: promoting direct cancer cell death and driving specific anti-tumoral immunity. In an attempt to develop a second-generation Accum^®^ molecule with enhanced therapeutic potency, a series of 7 variants were generated by interchanging the bile acid moiety of the molecule. Assessment of the in vitro killing potency of these variants identified the CDCA-SV40 dimer variant as a lead compound with consistent pro-killing potency on all tested murine and human cancer cell lines. The use of CDCA-SV40 not only improved the IC_50_ dose compared to the parent molecule, but it also retained most of the innate properties observed with Accum^®^ (Graphical Abstract).

Compared to Accum^®^, AccuTOX^®^ modulates a larger number of genes with specific activation of oxidative phosphorylation and ROS production amongst other pathways (Suppl Figs. 3–4). These resulting effects could be also linked to the observed inhibition of the TP53 pathway, an important component of cellular integrity, which if impaired, could result in amplified intracellular toxicity and a blockade in the cell’s ability to repair genotoxic effects affecting DNA [20]. It is however unclear if AccuTOX^®^ triggers these processes simultaneously or in tandem. Of note, only NAC pre-treatment could reverse the pro-killing activity of AccuTOX^®^ suggesting a possible direct binding of this antioxidant to AccuTOX^®^ via the cysteine residue separating the bile acid from the peptide sequence. Interestingly however, blocking mitochondrial ROS had no visible effect on cell death indicating that AccuTOX^®^-induced ROS production is potentially generated by other sources. Indeed, NADPH oxidases are found both on the cell surface or within the endosomal lumen (possibly due to membrane invagination during endocytosis to form endosomal structures) [21]. Therefore, AccuTOX^®^ could possibly bind and activate intra-endosomal NADPH oxidases resulting in ROS build-up that could impair both endosomal structures and cell function integrity.

Transcriptomic analysis underscored yet another salient observation triggered by AccuTOX^®^: antigen presentation. In fact, cancer cells have developed various means to bypass cytotoxic T lymphocytes (CTLs) responses including the downregulation of cell surface MHCI molecules [22]. In such context, the function of CTLs developed to recognize specific peptides in the context of MHCI is impaired, which would allow tumors to continue growing while amplifying other escape mechanisms such as the production of immune-suppressive, angiogenic factors and/or the recruitment of suppressive cells [23]. Interestingly, low dose AccuTOX^®^ increases both cell surface levels of H2-K^b^ as well as genes involved in antigen presentation as shown with the EG.7 tumor model. This is not only relevant in the context of using AccuTOX^®^ as an anticancer molecule, where ICD and antigen presentation could synergize in promoting anti-tumoral immunity, but it also suggests that AccuTOX^®^ could be used as an ex vivo agent to potentially enhance antigen presentation or cross-presentation in host-derived antigen presenting cells. Additional studies are therefore warranted to further investigate this novel function or to develop additional AccuTOX^®^ variants endowed with better antigen presentation capabilities.

## Conclusions

Although several immunomodulatory therapies such as ADCs and/or ICI have greatly enhanced anti-tumoral immunity, a large subset of patients do not respond effectively to these treatments or experience cancer relapse after an initial response [24, 25]. Thus, there is room for improving anti-tumoral responses using modalities that promote the killing of cancer cells while "flagging" them to the immune system. Here lies the importance of our proposed therapy as AccuTOX^®^ disrupts various intracellular processes leading to a chaotic dysregulation of normal cellular functions while promoting elements related to antigen presentation. For instance, AccuTOX^®^ not only elevates intracellular ROS levels promoting genotoxic effects, but it is potentially capable of blunting the endosomal transport mechanism while triggering a form of cell death as a danger signal recognized by pro-inflammatory immune cells. Although AccuTOX^®^ represents an injectable second-generation Accum^®^-based anti-cancer therapeutic, it poses great potential to serve as a possible payload to other cancer-specific mAb or ADCs as a means to amplify their therapeutic potency.

### Supplementary Information

Supplementary Material1.

## Data Availability

The datasets used and/or analysed during the current study are available from the corresponding author on reasonable request.

## Abbreviations

ADC: Antibody–drug conjugate
ANOVA: One-way analysis of variance
CTL: Cytotoxic T-Lymphocyte
CTLA-4: Cytotoxic T-Lymphocyte Associated Protein 4
CD: Cluster of differentiation
DAMPs: Damage-associated molecular patterns
DC: Dendritic cell
CM: Conditioned media
CTLA4: Cytotoxic T-lymphocytes associated protein 4
Cyt-C: Cytochrome C
DHE: Dihydroethidium
ICD: Immunogenic cell death
ICI: Immune-checkpoint inhibitor
IP: Intraperitoneal
IT: Intra-tumoral
HER-2: Human epidermal growth factor receptor-2
MAB: Monoclonal antibody
MSCs: Mesenchymal stromal cells
NAC: N-Acetylcysteine
NLS: Nuclear localization signal
OVA: Ovalbumin
PD-1: Programmed death 1
ROS: Reactive oxygen species
SC: Subcutaneous
