# Supplementary material for: Local delivery of accutox® synergises with immune-checkpoint inhibitors at disrupting tumor growth
Source: J Transl Med. 2024 Jun 3;22:532. doi: 10.1186/s12967-024-05340-2 (PMC11149357; doi:10.1186/s12967-024-05340-2)
Supplement: Supplementary file 1 — Supplementary Material1. [file 12967_2024_5340_MOESM1_ESM.docx]

**Supplementary Figure 1. The effect of Accum^®^ variants on different cancer cell lines**. **A)** Flow-cytometry analysis of Annexin-V^+^ events conducted on various murine and human cancer cell lines in response to different Accum^®^ variants (used at a concentration of 95 μM). The original Accum^®^ molecule is depicted by the gray filled histogram. **B)** Similar to panel A except that EL4 were treated with modified versions of the CDCA-SV40 molecule selected from the screening conducted on the cancer cell lines shown in panel A.

**Supplementary Figure 2.** **Cell surface expression of immune-checkpoint expression on the used cancer cells.** **A-C**) Flow-cytometry analysis of immune-checkpoint expression on the surface of EL4 (A), E0771 (B) and B16 (C) cells. Isotype controls are shown by filled gray histograms.

**Supplementary Figure 3. Molecular characterization of AccuTOX^®^-treated EL4 cells.** Displaying the top upregulated Reactome processes identified through an overrepresentation test of genes up or downregulated by AccuTOX^®^, with an adjusted p-value (False Discovery Rate) set at 5%.

**Supplementary Figure 4. Molecular characterization of AccuTOX^®^-treated EL4 cells.** Displaying the top downregulated Reactome processes identified through an overrepresentation test of genes up or downregulated by AccuTOX^®^, with an adjusted p-value (False Discovery Rate) set at 5%.
